# Clinical and radiological features of knee osteoarthritis in patients attending the university hospital of Kinshasa, Democratic Republic of Congo

**DOI:** 10.11604/pamj.2019.34.29.11283

**Published:** 2019-09-13

**Authors:** Adolphe Lukusa, Jean-Jacques Malemba, Pierrot Lebughe, Pierre Akilimali, Jean-Marie Mbuyi-Muamba

**Affiliations:** 1 Internal Medicine Unit, Ngaliema Hospital, Kinshasa, DR Congo; 2 Rheumatology Unit, University Hospital of Kinshasa, Kinshasa, DR Congo; 3 Public Health School of Kinshasa, Kinshasa, DR Congo

**Keywords:** Knee, osteoarthritis, Congolese, Kinshasa

## Abstract

**Introduction:**

The aim of the present study was to describe the clinical and radiological features of knee osteoarthritis in Congolese outpatients attending the University Hospital of Kinshasa (UHK).

**Methods:**

A cross-sectional study was performed in the rheumatology unit of the UHK from January to August 2012. Patients were consecutively recruited. The diagnosis of Osteoarthritis (OA) was based on the criteria of the American College for Rheumatology. Demographic, clinical and x-rays data were collected. The X-rays severity was assessed according to Kellgren and Lawrence's method.

**Results:**

1049 patients attended the Rheumatology unit of the UHK during the study period. An accurate diagnosis was reported for 839 patients, of whom 376 (44.8%) suffered from OA. Knee OA was diagnosed in 118 patients (31.4% of all OA patients). 101 patients accepted to be included in the study, 78 women (77.2%) and 23 men (22.8%). Their average age was 58.9 ± 10 years. A body mass index (BMI) ≥ 25kg/m^2^ was observed in 68 patients of whom 28 were obese (BMI ≥ 30kg/m^2^). The main symptoms were a mechanical pain (100%), swelling (40.6%), crepitus (79.2%) and mobility reduction (X%). Knee deformities were observed in some patients. At baseline, radiological damages > stage 2 of Kellgren-Lawrence were found in 70 patients.

**Conclusion:**

Knee OA is a common disease among outpatients who attend the unit of Rheumatology of the UHK. Its clinical profile is the same as what is reported in the literature. Obesity and skeletal abnormalities are encountered in the majority of patients.

## Introduction

Osteoarthritis (OA) is the most common joint disease but it remains one of the least studied rheumatic diseases in Africa [1]. Knee OA affects a large number of people, especially among people of 50 years and over. Its progression is slow and irreversible, and often leads to serious disability problems. In 1986, the American College of Rheumatology (ACR) proposed criteria for the diagnosis of the knee OA [1]. But there is no international agreement on the criteria to be used in epidemiological and clinical studies or on methods of monitoring OA patients [2]. The prevalence of knee OA in different studies depends on diagnostic criteria (clinical or radiological) and on the characteristics of the studied populations [3]. Knee OA concerned 2.9% of women aged between 45 and 65 years in the Chingford Study [4]. In the Framingham study (US), this prevalence was 6.1% in adults patients aged more than 30 years and 9.5% in old people [5]. In France, the prevalence of symptomatic knee OA is approximately 9% of people over 40 years [6]. In sub-Saharan Africa, some hospitalized based studies have been conducted on knee OA [7-14]. Ouédraogo *et al*. in Burkina Faso [7], and Eti *et al*. in Ivory Coast [8], observed that more than 80% of patients were women. In the Democratic Republic of Congo (DRC), knee OA is frequent among patients attending the University Hospital of Kinshasa (UHK), as reported in previous studies [13, 14]. With the aging population and the increasing prevalence of obesity, the number of people suffering from OA is expected to grow over next decades. This disease is an important public health problem and is of interest for researchers in rheumatology, taking into account its functional disorder and impact [15, 16]. The objective of the present study was to describe the clinical and radiological profile of knee OA in Congolese patients attending the UHK.

## Methods

A cross-sectional study was conducted in outpatients who attended the rheumatology unit of the UHK during the period from 3 January to 28 August 2012. Patients suffering from knee OA were recruited according to the criteria of the American College for Rheumatology (ACR) [1]. The following patients' data were noted: age, sex, BMI, joint complaints (pain, swelling and limitation of movement), the length of lower limbs, knee deformities, knee X-rays damages and the disease duration. The X-rays severity was assessed according to the score of Kellgren and Lawrence. This score comprises the following four stages: 1. Osteophytes of uncertain signification, 2. Osteophytes without joint space modification, 3. Osteophytes with joint space narrowing, 4. severe joint space narrowing and subchondral sclerosis. The tenderness level was assessed by the patient himself through a visual analog scale (VAS) ranged from 0 (no pain) to 100 mm (unbearable pain). Statistical analyses were performed using SPSS 18.0 software. Data were expressed as average and proportion (%). The t-Student test was used to compare averages. The analysis of variance was used to look for the association between the VAS values and the disease radiological stages. A p value < 0.05 was considered to be statistically significant. The present study was approved by the ethic committee of the Kinshasa's University. The consent of participants was obtained. Data were collected and analyzed anonymously.

## Results

**Figure 1:**
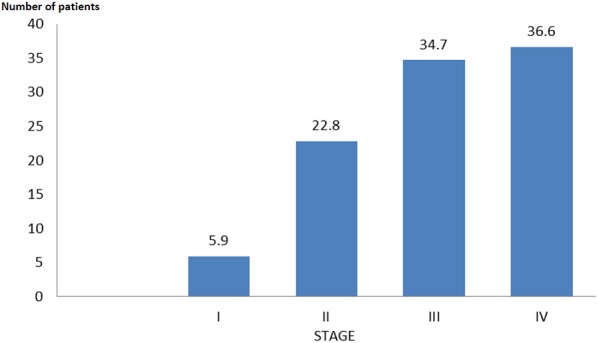
Classification of knee OA patients (n = 101) according to Kellgren - Lawrence score

One thousand and forty-nine (1049) patients attended the Rheumatology Unit of the UHK between 3 January and 28 August 2012. An accurate diagnosis was reported for 839 patients, of whom 376 (44.8%) suffered from OA. Knee OA was diagnosed in 118 patients (31.4% of all OA patients). 15 patients stopped their follow-up and 2 refused to be included. 101 patients were therefore included in the present study, 78 women (77.2%) and 23 men (22.8%). The sex ratio M/F was 0.3. Their average age was 58.9 ± 10.0 years. 93 patients (92%) were more than 45 years old. Table 1 represents the distribution of patients suffering from the knee OA according to their age and sex. It shows that 67 patients (2/3 of patients) are aged between 50 and 69 years. Table 2 reports the anthropometric characteristics of the patients. The body mass index (BMI) was significantly higher in females (p = 0.02) than in males. The peak incidence was in the range of 25 to 29.99 kg/m^2^. We can see that 2/3 of patients were either overweight (BMI: 25-29.99 kg/m^2^) or obese (BMI ≥ 30 kg/m^2^). Obesity was encountered in 32% of women (25/78) and 13.6% of men (3/21). The OA symptoms encountered in the present study are summarized in Table 3. The pain and crepitus were the main manifestations. The duration of the morning stiffness was less than 20 minutes. The following X-rays modifications were observed: osteophytes in all patients, joint space narrowing in 72 patients (71.3%) and subchondral osteosclerosis in 37 patients (36.6%). The distribution of patients according to the radiological score of Kellgren- Lawrence is reported in Figure 1. This figure shows that more than 70% of patients were at least stage III. Table 4 shows the locations of the knee X-rays lesions in the present study. We note that patellofemoral involvement was the most frequent. Table 5 indicates that there was no association between the pain intensity at inclusion and the stage of X-rays abnormalities, with a variance ratio of 1.74 and a p-value of 0.2.

**Table 1:** Distribution of knee OA patients according to their age and sex (n=101)

Age (yr)	Male (n=23)	Female (n=78)	All (n=101)
30-39	1	3	4
40-49	1	12	13
50-59	8	23	31
60-69	8	28	36
70-79	5	12	17

**Table 2:** Anthropometric characteristics of knee OA patients

Characteristics		Sex		*p*
	All patients	Female	Male	
Weight (kg)	74 ± 13.5	74.8 ± 14.6	71.4±8.7	0.178
Height (cm)	163 ± 21	162.6 ± 6.4	165.4 ± 6.3	0.035
BMI (kg/ m^2^ **)**	27.9± 5.4	28.4 ± 5.8	26.2 ± 3.8	0.02
< 18.5 kg/ m^2^ (%)	1	1	1	
18.5-24.99 kg/m^2^ (%)	31	24	7	
25-29.99 kg/m^2^ (%)	40	12	28	
≥ 30 kg/m^2^ (%)	28	25	3	

**Table 3:** Symptoms observed in knee OA patients at the UHK (n = 101)

Symptoms	N (%)
Knee pain	101 (100)
Swelling	41 (40.6)
Movement limitation	28 (27.7)
Morning stiffness	26 (25.7)
Axial deviations	37 (36.6)
Limping	30 (29.7)
Quadricipital amyotrophy	29 (28.7)
Crepitus	80 (79.2)

**Table 4:** Locations of X-rays damages of the knee’s OA in the present study

Locations	N (%)
IFTA*	49 (28.3)
EFTA **	20 (11.6)
IFTA* or EFTA**+ FPA***	40 (23.1)
FPA***	64 (36.9)

* IFTA: internal femorotibial arthritis

** EFTE: External femorotibial arthritis

*** PF: Patellofemoral

**Table 5:** The pain intensity in knee OA patients in relation with radiological stage

X-rays stage	VAS/pain	P = 0.2
I	58.3±13.3	
II	53.5±11.9	
III	47.7±15.3	
IV	53.1±12.8	

VAS: Visual analogue scale

## Discussion

The aim of the present study was to describe clinical and radiological features of knee OA in Congolese patients who attended the unit of Rheumatology of the University Hospital of Kinshasa. The main finding was that the knee OA was a common cause of consultation since it concerned 31.4% of OA patients. OA was diagnosed in 44.8% of all patients who attended the rheumatology unit. Knee OA was more frequent in females and in patients over 50 years old. Overweight and skeletal deformities were also involved in the onset of the disease. The high frequency of OA is in agreement with published data which show that OA is the most common [1, 17] rheumatic disease. The knee OA is very common in Congolese patients as reported in previous studies by Bwanahali *et al.* [13] and by Malemba and Mbuyi-Muamba [14]. A high incidence of knee OA was also observed in Arab people [18]. Genetic factors may play a role in the location of OA [19, 20]. Some studies conducted in multiracial areas such as USA and Hong-Kong supported the possible role of genetic factors. For example, fingers and hip OA are common in whites but rare in blacks [19, 20]. It is clear that the finger joints are most commonly used in daily activities in all populations and races. Therefore the main reason for the extreme rarity of fingers OA in blacks would be genetic. The role of genetics has also been reported by a British study of twins suffering from OA. It concluded that the influence of genetic intervened to 39-74% (depending on location) in the pathogenesis of OA [21]. Chitnavis in the USA described a lot of cases of familial knee OA [22]. Environmental factors such as obesity and daily activities are also involved in the frequency of knee OA. The role of obesity in the development of knee OA is well known [23, 24]. In the present study, overweight and obesity were observed in 68% and 28% of patients respectively. These conditions may aggravate OA because knees participate in supporting the body weight. Many studies have demonstrated a significant relationship between BMI and the risk of developing the knee OA [13, 25, 26]. Each kg/m^2^ in excess over a BMI of 27 kg/m^2^ increases the risk by 15%. The average age of patients in this study was 58.9 ± 10.0 years. It's known that advanced age is a risk factor for OA [6, 7]. Looser *et al.* [26] postulated that factors such as age-related alteration of cell and tissue function, sarcopenia, loss of proprioception and increased ligament laxity, combined with other factors such as obesity, traumatism and genetic factors lead to the occurrence of OA. The predominance of the female sex observed in the present study was also reported in several studies [6-8, 15, 16, 27-30]. The high frequency of obesity in women and menopause may play a role in this female predominance. Two studies performed in West Africa also observed that obesity was more common in females than males [7,8]. In addition to that postmenopausal statue may be as a potential risk factor for OA, but this notion must be considered with caution [31-33]. The role of abnormal musculoskeletal static in the onset of OA is well known [34, 35]. These abnormalities induce an unequal distribution of mechanical stresses on the articular surface. But OA can also lead to joint deformities such as genu varum or valgum, and other abnormalities, depending on the site of the disease. It is therefore difficult to confirm that the joint deformities encountered in this study appeared before the OA and not the contrary. A longitudinal follow-up may help to answer such a question. The fact that 70% of patients presented with advanced X-rays damages may be explained by the long delay between the onset of symptoms and the first consultation. The poverty of Congolese people, the use of self-medication or traditional medicine and the lack of information (on the existence of specialized rheumatology consultations) may explain the delay before consultation. The lack of association between the pain intensity (VAS) and the stage of X-ray abnormalities shows the discrepancy which may exist between the OA symptoms and its X-rays profile.

## Conclusion

Knee OA is a common disease in the service of Rheumatology of the University Hospital of Kinshasa. It affects mainly women more than men and people over 50 years. Its clinical feature is dominated by a knee pain, crepitus. At diagnosis, radiological damages are at an advanced stage, meaning that patients show up late. Obesity and skeletal abnormalities may play a role in the onset of the OA.

### What is known about this topic

- Knee osteoarthritis is a common rheumatic disease;
- It's a frequent cause of functional handicap;
- Some factors are implied in the occurrence of knee osteoarthritis such as age, obesity and joint deformities.

### What this study adds

- The present study is the first in DRC which concerns particularly knee OA;
- It shows that knee OA has the same risk factors and clinical features as what is described in the literature, but at inclusion 70% of patients presented an advanced knee OA;
- This study shows that Congolese health system must be improved, so that patients can consult physicians a bit earlier.

## Competing interests

The authors declare no competing interests.
